# Associations of health status with subsequent blood donor behavior—An alternative perspective on the Healthy Donor Effect from Donor InSight

**DOI:** 10.1371/journal.pone.0186662

**Published:** 2017-10-19

**Authors:** Katja van den Hurk, Saurabh Zalpuri, Femmeke J. Prinsze, Eva-Maria Merz, Wim L. A. M. de Kort

**Affiliations:** 1 Department of Donor Studies, Sanquin Research, Amsterdam, The Netherlands; 2 Department of Sociology, VU University, Amsterdam, The Netherlands; 3 Department of Public Health, Academic Medical Center, Amsterdam, The Netherlands; Germany Red Cross, GERMANY

## Abstract

**Introduction:**

In donor health research, the ‘Healthy Donor Effect’ (HDE) often biases study results and hampers their interpretation. This refers to the fact that donors are a selected ‘healthier’ subset of a population due to both donor selection procedures and self-selection. Donors with long versus short donor careers, or with high versus low donation intensities are often compared to avoid this HDE, but underlying health differences might also cause these differences in behaviour. Our aim was to estimate to what extent a donor´s perceived health status associates with donation cessation and intensity.

**Methods:**

All active whole blood donors participating in Donor InSight (2007–2009; 11,107 male; 12,616 female) were included in this prospective cohort study. We performed Cox survival and Poisson regression analyses to assess whether self-reported health status, medication use, disease diagnosed by a physician and recently having consulted a general practitioner (GP) or specialist were associated with (time to) donation cessation and donation intensity.

**Results:**

At the end of 2013, 44% of the donors in this study had stopped donating. Donors in self-rated good health had a 15% lower risk to stop donating compared to donors in perceived poorer health. Medication use, disease diagnoses and consulting a GP were associated with a 20–40% increased risk to stop donating and a lower donation intensity, when adjusting for age, number of donations and new donor status. Both men and women reporting good health made on average 10% more donations.

**Conclusion:**

Donors with a “good” health status were less likely to stop donating blood and tended to donate blood more often than donors with perceived poorer health status. This implies that the HDE is an important source of selection bias in studies on donor health and this includes studies where comparisons within donors are made. This HDE should be adjusted for appropriately when assessing health effects of donation and donors’ health status may provide estimates of future donation behavior.

## Introduction

According to several studies, blood donation is associated with better health outcomes. A 33–88% lower risk of cardiovascular disease (CVD) has been reported in donors versus non-donors.[1, 2] Repeated blood donations reduce blood viscosity, thereby potentially lowering blood pressure and the risk of plaque rupture.[3, 4] It is also known that with a 500ml whole blood donation, up to 250mg of heme iron is lost.[5–7] This iron loss, which may reduce oxidative stress and the availability of iron for malignant cells, may have a protective effect against insulin resistance and atherosclerosis, and may as well protect against cancer.[6, 8–11]

**Fig 1:**
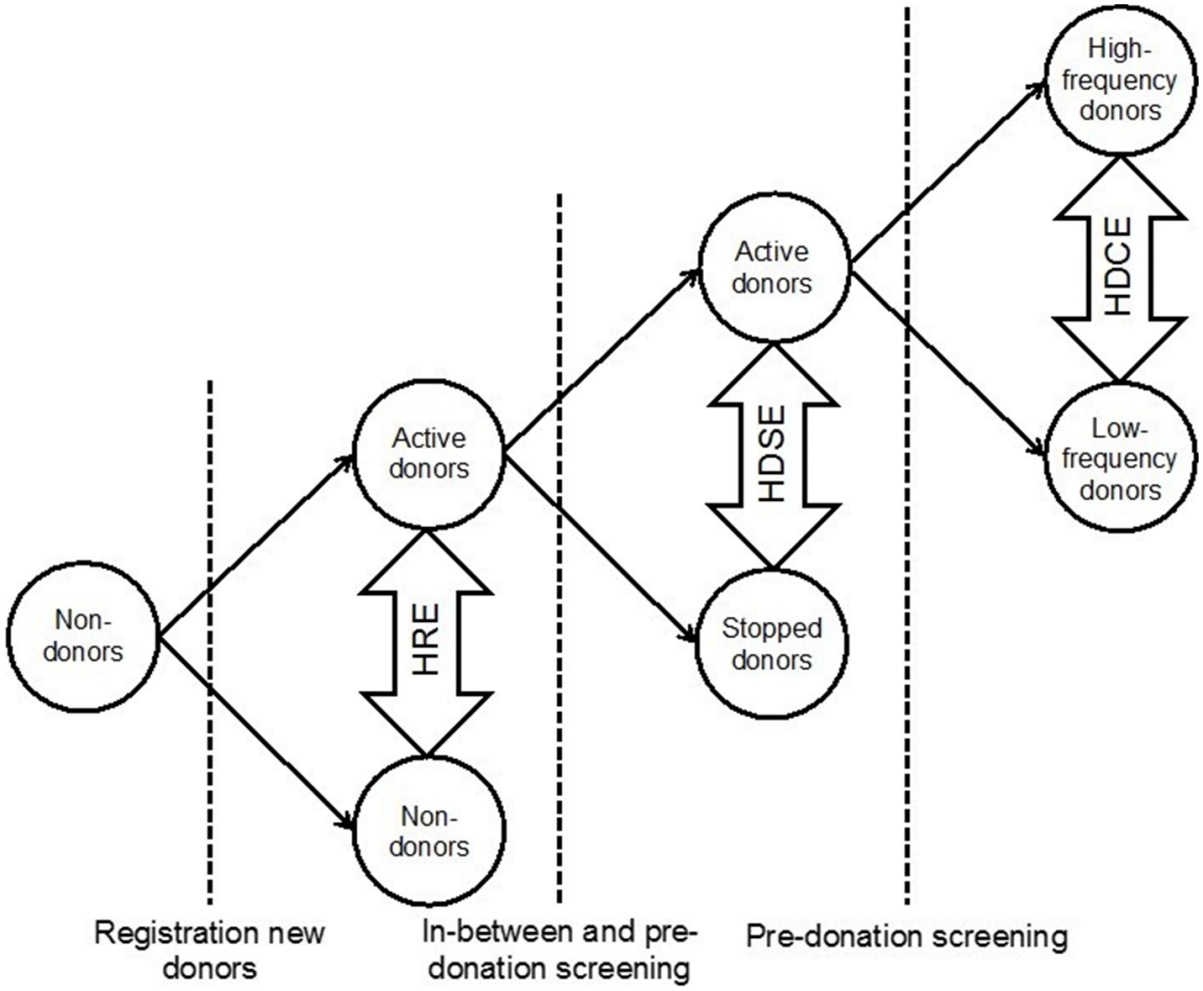
The Healthy Donor Effect in three stages. HRE = Healthy Registration Effect, HDSE = Healthy Donor Survivor Effect, HDCE = Healthy Donor Career Effect. (Adapted with permission from “Blood Donation and Cardiovascular Disease—Addressing the Healthy Donor Effect”, PhD thesis by K. Peffer, 1-12-2015. ISBN: 978-90-6464-933-2).

Previous studies that have addressed effects of blood donation on health have been inconclusive and contradictory, mainly because researchers have not been able to appropriately deal with selection bias: the so-called Healthy Donor Effect (HDE).[8, 12–16] The HDE refers to the fact that donors are a selected ‘healthier’ subset of the general population as they are subject to both donor selection procedures and self-selection. In light of these selection effects, the main challenge in donor health research is to distinguish whether health differences between donors and non-donors are due to health effects of donation; or due to donor selection. The HDE can be divided in three subtypes (Fig 1): the Healthy Registration Effect (HRE), the Healthy Donor Survivor Effect (HDSE), and the Healthy Donor Career Effect (HDCE).[15] The HRE refers to health differences between individuals who register to donate and those who do not, for instance because of prevalent disease, and is considered to be the largest of the three HDE subtypes.[15] The HDSE refers to comparisons of donors who continue to donate with those who stop, which can be due to temporary or permanent donor deferrals. The third subtype of HDE, the HDCE, applies to comparisons within active donors: high-frequency donors are compared to low-frequency donors, or donors with high to those with low lifetime numbers of donations.

The current line of thinking is that because of the relatively large HRE, effects of blood donation on health should be studied within the group of donors- comparing donors who donate more or less frequently; or with long versus short donor careers.[13, 17] We hypothesize that these approaches do not sufficiently deal with the HDE. A risk remains of attributing observed health differences to the effects of donation in spite of the fact that these differences were actually pre-existing and determined whether or not a donor continued to donate, or in- or decreased their donation frequency.[18, 19]

Donor InSight (DIS) is a cohort study aimed at gaining insight into the characteristics, health and motivation of blood and plasma donors throughout the Netherlands. DIS’ linkage to actual donation registry data provides the opportunity to link the health status of donors with their subsequent donation behaviour. This enables us to prospectively investigate to what extent the health status of donors is associated with the time to donation cessation and donation intensity.

## Methods

### Study population

Donor InSight (DIS) is a self-administered questionnaire study aimed at gaining insight into characteristics and motivation of the Dutch donor population.[20] Questionnaires were completed between April 2007 and April 2009 by a representative sample of 31,338 Sanquin donors (63% of those invited, 8% of the entire donor population of ~400,000 in 2009). Sanquin is the only organisation in the Netherlands authorised to supply blood products and donations are on a voluntary, non-remunerated basis. The Medical Ethical Committee Arnhem-Nijmegen in the Netherlands approved DIS and all participants gave their written informed consent.

DIS data have been linked to donation data as registered in the blood bank information system (PROGESA, MAK-SYSTEM International Group, France). PROGESA data include donation types, (e.g. whole blood or plasma), volumes, and dates of all donation attempts in the Netherlands. Donors must meet several criteria according to European guidelines in order to be eligible to donate.[21] They have to be between 18 and 70 years of age. Certain health conditions and high-risk behaviours can lead to temporary or permanent deferral from donation. Permanent deferrals lead to deregistration from the database.

For the current analyses, DIS participants who were active whole blood donors at the time of their participation in DIS were included (n = 23,723). This implied that they made at least one donation attempt within two years prior to their participation in DIS and were registered as whole blood donors at the time of their invitation to participate in DIS.

### Donation cessation and intensity

To determine whether a donor stopped donating or not, each donor’s donation attempts up until 31-12-2015 were extracted from PROGESA. Donors who made their last donation attempt more than two years prior to 31-12-2015, or more than two years prior to their 70^th^ birthday (the maximum age for donation in the Netherlands) were labelled as stopped donors. This definition is in line with international donor management criteria.[22] Time from participation in DIS to their last donation attempt was calculated in days. For those who were not labelled as stopped donors, time to their 70^th^ birthday (if applicable) or censoring (31-12-2013) was calculated in days.

To determine donation intensity after participation in DIS, each donor’s number of whole blood donation attempts within two years after their participation in DIS was counted.

If any reason to stop donating is known, this is recorded in PROGESA. We categorised all reported reasons to stop donating into 4 categories: practical reasons (opening hours, closing of locations, distance from a location, etc.), personal reasons (private circumstances), health/medical reasons (permanent deferral or self-withdrawal for medical reasons or health issues) and deceased/non-response/unknown reasons.

### Health status

Each donor’s perceived health status was assessed in the DIS questionnaire by five different questions:

1. Donors were asked to rate their accordance with the statement *`My health is excellent´* on a 5-point scale, ranging from fully disagree to fully agree. These answers were recoded into two dummy variables; ‘poor health’ ((fully) disagree, rated 1) versus neutral (rated 0), and ‘good health’ ((fully) agree, rated 1) versus neutral (rated 0) in the regression analyses. These two dummy variables are always jointly used in regression models, in order not to exclude subgroups of donors from analyses. Additional analyses with four dummy variables were checked but showed no different associations.
2. Secondly, donors filled in whether they currently used any medication (yes versus no) by completing an extensive list of medication types. For the present analyses, we assessed whether a donor used any type of medication, excluding supplements.
3. Furthermore, we asked whether a donor had any prevalent disease, excluding fertility problems, hemochromatosis, allergies and skin problems (yes versus no).
4. All donors were asked whether they consulted their general practitioner within three months prior to filling out the DIS questionnaire (yes versus no). Note that in the Netherlands, a general practitioner functions as a gate keeper to the more specialized part of the health care system.
5. Finally, donors were asked whether they consulted a medical specialist within 12 months prior to their participation in DIS (yes versus no).

### Confounders

Age was calculated at the time of participation in DIS. Current or prior smoking status was assessed by questionnaire. Baseline donation intensity was assessed by counting the number of whole blood donation attempts in the two years prior to participation in DIS. Donors who had their very first donor screening within the two years prior to participating in DIS, were considered to be new donors.

### Statistical analyses

Descriptive statistics were presented as means ± standard deviations (SD) or, in the case of a skewed distribution, as median (interquartile range), for men and women separately. Associations of perceived health status with donation cessation were analysed with Cox proportional hazards models. Results of these hazard models were expressed as hazard ratios (HR) with 95% confidence intervals (95% CI). These survival models relate the time that passes before donors stop donating to their perceived health status at the time of their participation in DIS. For example, they assess whether donors with a prevalent disease were more likely to stop donating at any point in time as compared to donors who did not have a disease. Associations of health status with donation intensity were analysed with Poisson regression models and the results expressed as relative risks (RR) with 95% CI. All analyses were done separately for men and women, mainly because men can donate more often than women in the Netherlands (5 versus 3 times per year), which impacts donation intensities. Furthermore, all analyses were adjusted for age, smoking status, number of previous donations and new donor status. Analyses were performed in SPSS 23.0 (IBM, New York, U.S.A.) and p values lower than 0.05 were considered statistically significant.

## Results

### Characteristics of the study population

The study population comprised of a total of 23,723 blood donors. Men comprised 47.8% of the total population, with a mean age of 49 years, compared to 43 years in women (Table 1). Both men and women had approximately the same percentage of current smokers (17% and 16% respectively).

**Table 1:** Baseline characteristics of whole blood donors in the Donor InSight (DIS) population.

	Men (n = 11,107)	Women (n = 12,616)
**Age at DIS participation (years)**	49 ± 12	43 ± 13
**BMI (kg/m**^**2**^)*	25.7 ± 3.1	24.9 ± 4.0
**Current smoker**	1848 (17%)	2053 (16%)
**Former smoker**	4481 (40%)	4343 (34%)
**Baseline donation intensity**	5 (3–7)	3 (2–5)
**1–3 donations**	36%	57%
**4–6 donations**	35%	43%
**More than 6 donations**	29%	0%
**New donor status**	863 (7.8%)	1815 (14.4%)

Continuous data are shown as mean ± standard deviation, donation intensity as median (interquartile range).

*Data from Progesa were assessed as of 31-12-2009, unless stated otherwise.

With regards to the baseline donation intensity among the study participants, median numbers of donations in the two years prior to participation in DIS were 5 for men and 3 for women (Table 1).

Thirty-six percent of men and 57% of women had a baseline donation intensity of 1–3 donations (Table 1). A smaller group of donors belonged to the donation intensity category of 4–6 donations (35% and 43% in men and women, respectively). Twenty-nine percent of men donated more than 6 times and none of the women did. The latter is caused by Sanquin’s policy that women can only donate up to three times per year, while men are allowed to donate up to five times per year.

There was no difference in the weekly physical activity between men and women as measured by at least 150 minutes (or 75 intensive minutes) of physical activity (sports, biking) per week, excluding walking. 75.9% amongst women, and 76.7% male donors reported yes to the above definition of high weekly activity.

### Health status

On the self-rated DIS questionnaire, 44% of all men and 45% of all women strongly agreed on being in excellent health (Table 2). On the other end of the spectrum, 2% of the men and 1% of the women strongly disagreed on being in excellent health. Twenty percent of men and 17% of women reported using medication at their participation in the study. Other reported health status indicators in men and women were a diagnosed disease (38% and 37%, respectively), a GP consultation in the past 3 months (26% and 34%) and consultation of a specialist in the past 12 months (17% and 21%).

**Table 2:** Health status of whole blood donors at their participation in Donor InSight (DIS, 2007–2009).

		Men (n = 11,107)	Women (n = 12,616)
Excellent health*	Strongly agree	4864 (44%)	5720 (45%)
	Agree	4667 (42%)	5077 (40%)
	Neutral	1033 (9%)	1138 (9%)
	Disagree	340 (3%)	467 (4%)
	Strongly disagree	162 (2%)	175 (1%)
Current Medication use**		2214 (20%)	2126 (17%)
Diagnosed disease***		4225 (38%)	4662 (37%)
**Consulted GP past 3 months**		2894 (26%)	4241 (34%)
**Consulted specialist past 12 months**		1878 (17%)	2620 (21%)

Numbers of donors (n(%)) are shown, based on self-report data from the DIS questionnaire.

*self-rated health on a 5-point scale,

** excl. supplements,

*** excl. fertility problems, hemochromatosis, allergies and skin problems.

### Health status and donation cessation

Overall, 46% of the women and 36% of the men had stopped donating before 31-12-2013, or before becoming 70 years of age (Table 3). Among men, 32% stopped donating due to health or medical reasons, and 14% because of personal reasons. The main documented reasons among women were health or medical (25%) and personal (17%). For almost half of the stopped donors (47% of men, 50% of women) reasons were unknown.

**Table 3:** Outcomes among the donors during follow-up.

		Men (n = 11,107)	Women (n = 12,616)
Stopped donating*		3965 (36%)	5785 (46%)
	Practical reasons	272 (7%)	426 (7%)
	Personal reasons	545 (14%)	990 (17%)
	Health/medical reasons	1281 (32%)	1470 (25%)
	Deceased/non-response/unknown reasons	1867 (47%)	2899 (50%)
Follow-up donation intensity**	0	921 (8%)	1649 (13%)
	1–3	3395 (31%)	5574 (44%)
	4–6	3382 (30%)	5307 (42%)
	>6	3409 (31%)	86 (1%)

*Numbers of donors (n(%)) are shown who stopped donating before 31-12-2013, or before becoming 70 years of age.

**Numbers of donors (n(%)) are shown with 0, 1–3, 4–6 or >6 whole blood donation attempts in the two years after their participation in DIS.

**Fig 2:**
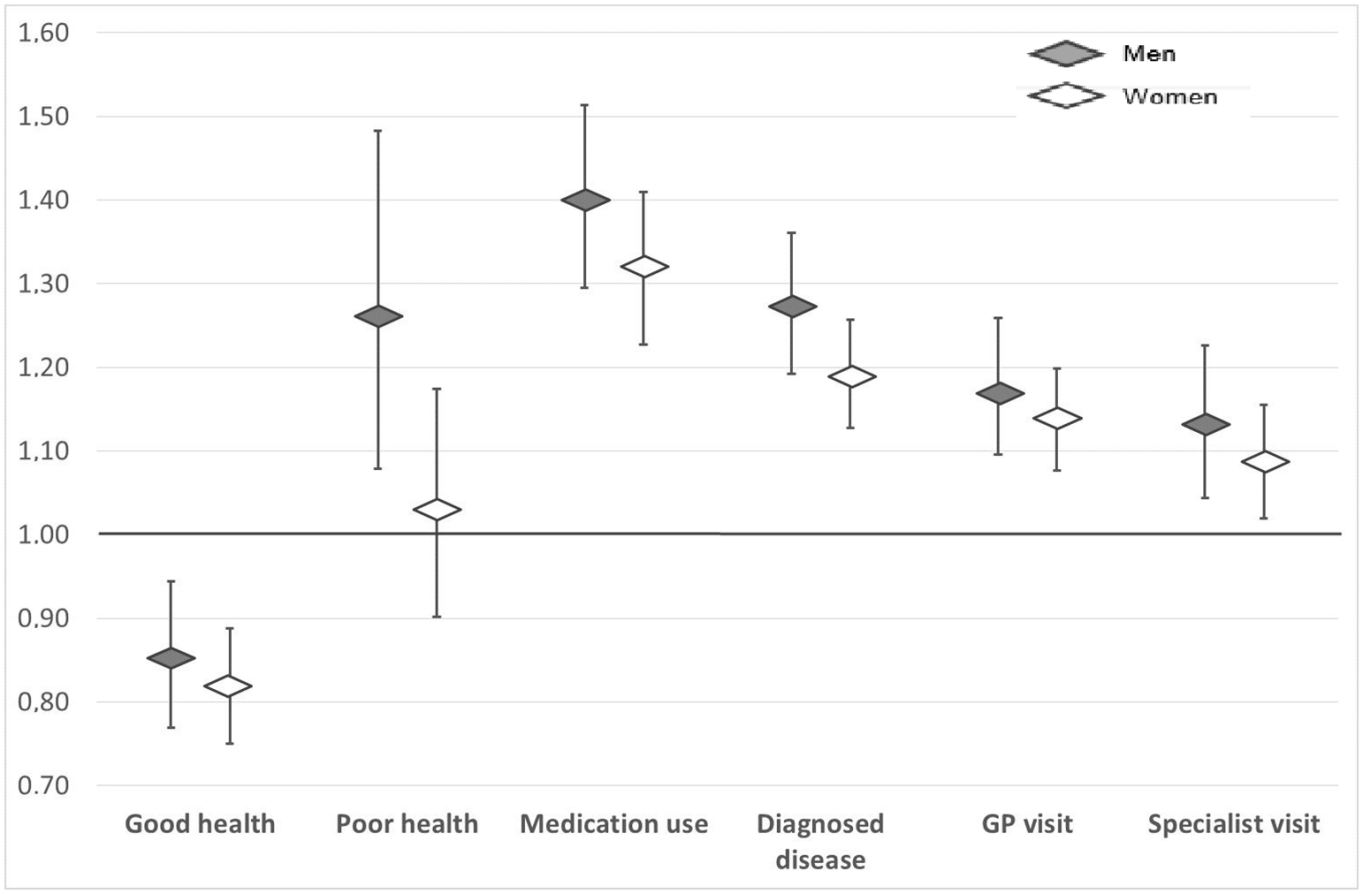
Associations of self-reported health status with time to donation cessation (hazard ratios and 95% confidence intervals), adjusted for age, smoking, baseline donation intensity and new donor status; for men (gray) and women (white). Time from participation in DIS to donation cessation or censoring (31-12-2013) was calculated in days.

Among men, donors with self-reported good health had a 15% lower risk to stop donating at any time during follow-up as compared to men with a normal health status after adjusting for age, smoking and baseline donation intensity (HR 0.85; 95% CI 0.77–0.94, Fig 2). Men with poor health (HR 1.27; 95% CI 1.08–1.48), on medication (HR 1.40; 95% CI 1.30–1.51), diagnosed with a disease (HR 1.27; 95% CI 1.19–1.36), a GP consultation in the past 3 months (HR 1.17; 95% CI 1.10–1.26) or consultation of a specialist in the past year (HR 1.13; 95% CI 1.04–1.23) all had a higher risk to stop donating at any time during follow-up.

In women, donors in perceived good health (compared to women with a perceived normal health status) were also less likely to stop donating (HR 0.81; 95% CI 0.75–0.88). Women with medication use (HR 1.32; 95% CI 1.23–1.41), diagnosed with a disease (HR 1.19; 95% CI 1.12–1.26), a GP consultation in the past 3 months (HR 1.14; 95% CI 1.07–1.20) or consultation of a specialist in the past year (HR 1.09; 95% CI 1.02–1.15) all had a higher risk to stop donating at any time during follow-up. Women with a perceived poor health status did not have a statistically significant higher risk to stop donating than women reporting a normal health status.

### Health status and donation intensity

Table 3 shows that, after following up the study participants, 31% of the men and 44% of the women had a donation intensity of 1–3 donations, 30% and 42%, respectively, had a donation intensity of 4–6 donations, and 31% and only 1%, respectively had a donation intensity of more than 6 donations.

After adjustment for age, smoking, baseline donation intensity and donor status, men with a perceived good health status made on average 10% more donations in the two years following their participation in DIS, compared to men with a perceived normal health status (RR 1.10, Table 4). Among women, a good health status was associated with a 9% higher donation intensity (RR 1.09).

**Table 4:** Associations of self-reported health status with donation intensity—Relative risk (95% confidence intervals).

	Men		Women	
	Crude	Adjusted*	Crude	Adjusted*
**Good health**	1.13 (1.10–1.17)	1.10 (1.07–1.14)	1.16 (1.11–1.20)	1.09 (1.06–1.14)
**Poor health**	1.01 (0.96–1.07)	1.04 (0.99–1.09)	0.97 (0.91–1.03)	0.97 (0.91–1.03)
**Medication use**	0.97 (0.95–0.99)	0.94 (0.91–0.96)	1.02 (0.99–1.05)	0.95 (0.93–0.98)
**Diagnosed disease**	0.98 (0.96–0.99)	0.96 (0.94–0.98)	0.99 (0.97–1.01)	0.95 (0.93–0.97)
**Visited GP past 3 months**	0.90 (0.88–0.92)	0.93 (0.91–0.95)	0.92 (0.89–0.93)	0.94 (0.92–0.96)
**Visited specialist past 12 months**	0.89 (0.87–0.91)	0.94 (0.92–0.97)	0.87 (0.85–0.89)	0.93 (0.90–0.96)

Poisson regression models for associations of self-reported health status with donation intensity; the number of whole blood donation attempts within two years after participating in Donor InSight.

*Adjusted for age, smoking, baseline donation intensity and new donor status.

Among male donors, the use of medication (RR 0.94); a disease diagnosis (RR 0.96); a GP consultation in the past 3 months (RR 0.93) or consultation of a specialist in the past year (RR 0.94) were all associated with a lower donation intensity. Similar effects were seen among women: the use of medication (RR 0.95); a disease diagnoses (RR 0.95); a GP consultation in the past 3 months (RR 0.94) or consultation of a specialist in the past year (RR 0.93) also indicated a lower donation intensity.

## Discussion

In our study among a Dutch cohort of whole blood donors, we found that the self-reported health status of both male and female donors impacted their subsequent blood donation behaviour. Donors with a “good” health status were less likely to stop donating blood and tended to donate blood more often. Donors on medications, with a disease diagnosis and who reported recent consultations of their GP or a specialist (as indicators of a relatively poorer health status) showed a tendency to either donate blood with a lower intensity or stop donating all together. We also observed that main documented reasons to stop donating were health or medical.

We conducted our study to better understand the impact of donor health on donation behaviour in whole blood donors. This information provides a quantified estimate of the HDE when assessing health effects of donation by comparing subgroups of donors. The study is unique because of its prospective rather than cross-sectional design, which allows us to isolate the HDE from health effects caused by donation. The next logical step would be to investigate how the act of blood donation affects donor health, thereby properly adjusting for this HDE.

The most important limitation of the study is that health status was only assessed by a questionnaire, and not by more objective measures. Nonetheless, we expect that perceived health (because of self-selection) and disease diagnoses (because of deferrals, assessed by asking about medication use and GP or specialist consultations) are most likely to influence donation behaviour. Another potential limitation of our study is the lack of response from 37% of the invited study population. This common source of bias mainly affects a study’s external validity and may thus hamper extrapolation to the entire donor population and to other donor populations. Nonetheless, as shown previously, the Donor InSight population is very similar to the overall Dutch donor population in terms of age, sex, haemoglobin levels and blood pressure and therefore not likely to be influenced by any major selection bias.[20, 23]

Any study looking into the health of blood donors; or into the impact of repeated blood donations on donor health are invariably biased due to this selection effect, or HDE.[15] The HDE routinely leads to either misinterpretation of study results or inconclusive results.[8] It has been previously shown that blood donors may be at a reduced risk of diseases, in-hospital admissions, in-hospital mortality[24] and lower risk of cardiovascular events.[1, 2] These seemingly beneficial effects might be a consequence of a healthier lifestyle and the continuous pre-donation check-ups that blood donors undergo in order to maintain their blood donor status.[24] Blood donors indeed represent a healthier subset of the general population[20]. The HDE has been elegantly quantified by Shehu et al. in their 2015 study.[25] They quantified the overall effect of the HDE on donation-related health outcomes. Their estimation was that up to 82% of the health differences between donors and non-donors were due to the HDE rather than to actual health effects of donation. By the means of a propensity score matching, Shehu et al. have demonstrated a way forward in an attempt to adjust for the HDE (both the HDSE and the HDCE).[25] The study also expresses its limitations on using self-reported health data as an indicator for the donor health status, as well as an inability to account for the motivational factors (for donation) among blood donors. Another recent study on blood donation and blood donor mortality found an inverse relationship between donation frequency and mortality.[26] The magnitude of this effect was lowered once the authors adjusted for the HDE, by introducing an indicator (in the model) for donors who had to stop donating because of the enforced upper age limit for blood donations. This study clearly shows the role of HDE in donor outcomes research and opens up a discussion to take into account this HDE across various age groups.

In occupational epidemiology, the well-described Healthy Worker Effect (HWE) draws parallels to the HDE in blood donor research.[27] This selection bias is characterized by lower relative mortality rates in an occupational cohort as compared to the general population, and occurs because relatively healthy individuals are more likely to gain employment and to remain employed. Far more efforts have been made in occupational research to account for this selection bias. They have ranged from comparing active workers to the general population; using active workers unexposed to certain detrimental occupational exposures, to comparing workers with a higher and a lower exposure.[28] Yet, the issue persists, because as with donor research and the HDE,[16] occupation epidemiology has also struggled to find the right reference group for comparisons of health benefits.[28]

To conclude, the HDE is an important source of selection bias in studies on donor health and this includes studies where comparisons within donors are made. This type of bias mainly concerns the internal validity of studies, potentially leading to false conclusions. We have prospectively shown that donors with “better” health indicators such as perceived health status and having consulted a physician less often, are less likely to stop donating and more likely to donate at a higher frequency. It therefore is essential to adjust for the HDE by taking donors’ pre-existing health status (and other health indicators) into account when investigating whether blood donation itself affects the health of blood donors. Asking donors about their perceived health status may also provide clues for donor retention. The impact of the donors’ perceived health on their donation behaviour therefore has clear implications for a) donor health research, where beneficial effects of donation might be overestimated and b) donor management, where a clear picture on donors’ health status may provide estimates of future donation behaviour.
